# Bystander Roles in Cyberbullying: A Mini-Review of Who, How Many, and Why

**DOI:** 10.3389/fpsyg.2021.676787

**Published:** 2021-05-28

**Authors:** Karina Polanco-Levicán, Sonia Salvo-Garrido

**Affiliations:** ^1^Programa de Doctorado en Ciencias Sociales, Universidad de La Frontera, Temuco, Chile; ^2^Departamento de Psicología, Universidad Católica de Temuco, Temuco, Chile; ^3^Departamento de Matemática y Estadística, Universidad de La Frontera, Temuco, Chile; ^4^Núcleo Científico y Tecnológico en Ciencias Sociales (LICSA), Universidad de La Frontera, Temuco, Chile

**Keywords:** cyberbullying, bystanders, cyberbystanders, roles, adolescents, literature review

## Abstract

Cyberbullying has progressively increased due to the massive use of the internet and social networks. Bystanders constitute the largest group, occupying a key role in the evolution of the cyberbullying situation and its consequences for the victim. Research shows different ways in which bystanders behave, suggesting different types of sub-roles associated with different study variables. The objective of this literature review is to identify and characterize the roles of bystanders in cyberbullying situations that involve adolescent students. To achieve this objective, a systematic search was carried out in the Web of Science, PubMed, and Scopus databases for articles published between 2015 and 2020, resulting in 233 articles. Articles were then selected by relevant title and summary. Subsequently, the inclusion and exclusion criteria were applied, resulting in a total of nine articles. The findings of this review allowed us to identify two to five types of bystanders, the largest type representing outsiders and the smallest type representing assistants of the aggressor. The identified types of bystanders are characterized for variables such as sex, age, previous experience, and empathy. The results are discussed considering the available theoretical and empirical evidence.

## Introduction

Technology and the internet have transformed society through a process of mutual interaction, allowing communication at any time and transcending territorial borders (Castells, 2014). Internet use has become widespread in adolescents, with 92.9% reporting having at least one social media account (Barry et al., 2017) and using an average of three different platforms daily (Vannucci and Ohannessian, 2019). Constant exposure to social networks and the internet raises concern about the possible negative effects on the well-being of adolescents, specifically due to phenomena such as cyberbullying, which is positively associated with online time (Lee and Shin, 2017; Shapka et al., 2018; Craig et al., 2020), and favored by the use of smartphones, which allow connection at any time and place (Martin et al., 2018).

Cyberbullying can be defined as a form of intentional harassment that is directed at a particular person, carried out through electronic and digital means and produced by an imbalance of power associated with greater development of technical skills when using the internet (Smith et al., 2013; Olweus and Limber, 2018). Additionally, anonymity is considered a significant risk factor in this phenomenon (Barlett et al., 2016, 2020). On the other hand, cyberbullying is considered a social phenomenon that can negatively affect victims, aggressors, and bully/victims, significantly increasing the risk of suicide (DeSmet et al., 2014, 2019; Hellfeldt et al., 2020; Kim et al., 2020).

Regarding the prevalence of cyberbullying, one in three adolescent participants reported having been a victim of cyberbullying (United Nations International Children's Emergency Fund (UNICEF), 2019). Research by Antoniadou et al. (2019) classified cyberbullying participants as bystanders (75%), bully/victims (11.2%), victims (8.2%), and aggressors (5.6%). Thus, the percentage of bystanders in cyberbullying situations was significantly higher than the other roles, making it relevant to deepen the understanding of bystander characteristics and behavior due to the impact their actions may have on the development of the situation and the experience of the victim and the aggressor.

### The Role of Bystanders in Cyberbullying and Its Relationship With Personal and Contextual Variables

At present, the role of bystanders in cyberbullying has received little attention, despite its relevance and conceptual differences with traditional bullying (Garaigordobil, 2017; Sarmiento et al., 2019). Specifically, Kozubal et al. (2019) reported that, when bystanders are exposed to a human face with an expression of sadness, they can modify their behavior and not reinforce cyberbullying. However, this is far from what happens on internet platforms since the facial expression of the victim is rarely seen.

Regarding the role of bystanders and sex, some studies support the hypothesis that adolescent women tend to show more supportive behaviors toward the victim compared to men (Machackova et al., 2016; Allison and Bussey, 2017; Campbell et al., 2017; Patterson et al., 2017), but there is also evidence that there are no significant differences between the sexes (Kozubal et al., 2019). Regarding bystanders and age, studies showed that there are no behavioral differences (Machackova et al., 2016; Campbell et al., 2017), although Pabian et al. (2016) reported that students had a less empathetic response 6 months after the first measurement, which could reflect a desensitization effect over time.

Regarding the role of the bystanders and their association with the socio-affective variables in cyberbullying, the presence of greater moral disengagement, less empathy, and a lower perception of responsibility and self-efficacy is reported, since bystanders do not see the emotional response of the victim, which interferes with their evaluation of the situation (Barlińska et al., 2018; Domínguez-Hernández et al., 2018; Knauf et al., 2018). On the other hand, there are contextual variables that affect the emergence and type of behavior displayed by bystanders in the face of cyberbullying, including degree of friendship, severity of the incident, actions of other bystanders (Domínguez-Hernández et al., 2018), and whether the situation is non-anonymous (You and Lee, 2019) or those situations that occur in private (DeSmet et al., 2014; Patterson et al., 2017). The last variable is linked to personal characteristics since adolescents with greater empathy will decide to intervene regardless of whether it is in private or in public (Wang, 2020).

Regarding the role of bystanders in traditional bullying, Salmivalli et al. (1996) and Salmivalli (2010) referred to different bystander types: assistants of the aggressor (join the aggressor), reinforcers of the aggressor (laugh or encourage the aggressor), outsiders (do not get involved in the situation), and defenders of the victim (support victims). This classification is used by the renowned anti-bullying program, Kiusaamista Vastaan (KiVa) (Salmivalli and Poskiparta, 2012), developed in Finland, which works with victims, aggressors, and bystanders. Similarly, within the role of the bystanders, specific types can be differentiated, making it important not to homogenize them since this would hinder the understanding of the implications that the different forms of behavior have on the other participants (Moxey and Bussey, 2019).

As a result of the above, it can be pointed out that there is not much knowledge about how bystanders behave in cyberbullying situations, making it necessary to continue generating data on the subject (Garaigordobil, 2017; Sarmiento et al., 2019; Íñiguez-Berrozpe et al., 2020). Along with this, the studies that address types of bystander behaviors denominate, group, and characterize them in different ways, making it difficult to understand the sub-roles and their characteristics. Therefore, the objective of this study was to identify and characterize the roles of bystanders in cyberbullying in adolescent students.

## Methods

**Figure 1:**
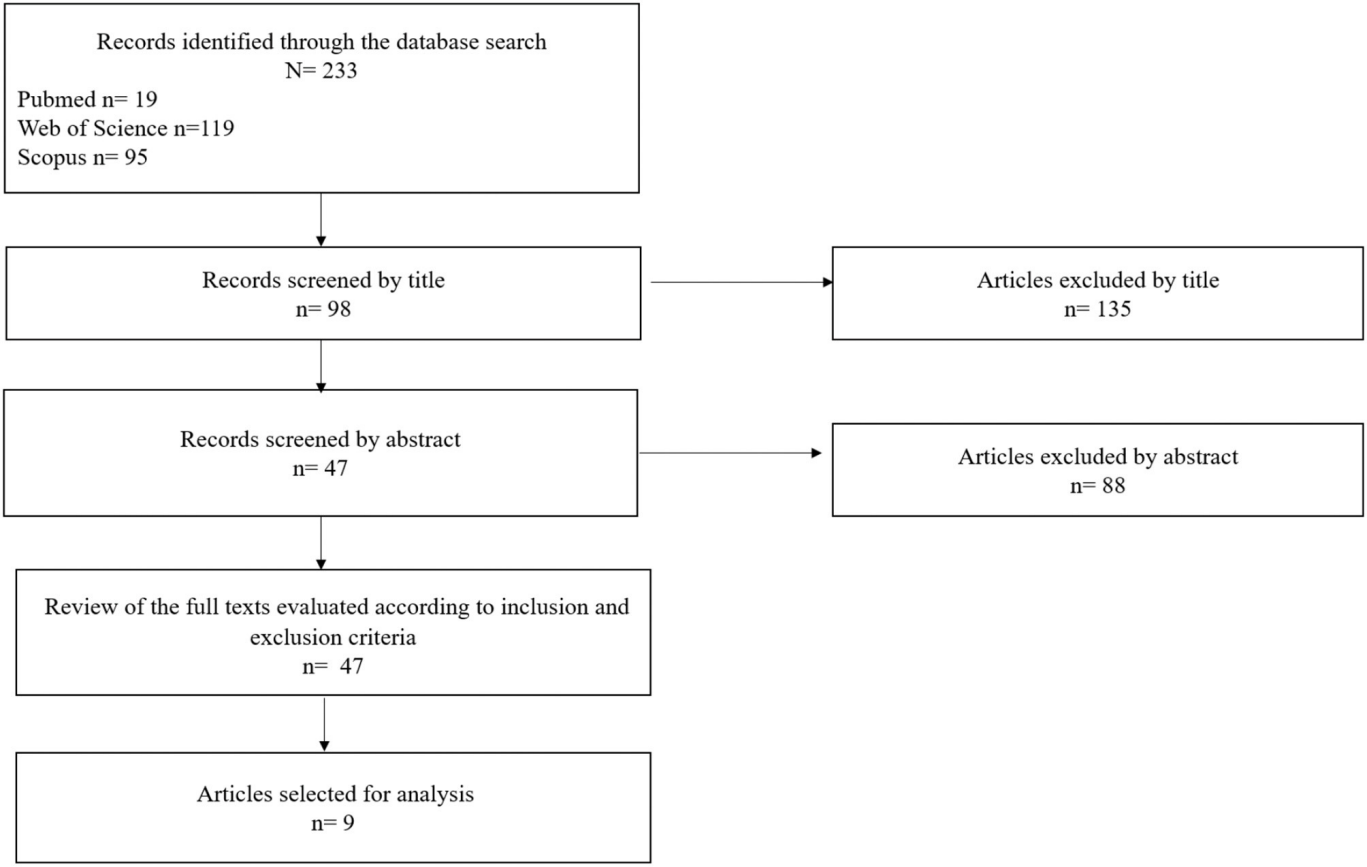
Flowchart of article selection.

This article reports the results of a systematic review of the different roles of bystanders in cyberbullying. A systematic literature search was carried out following the guidelines of Bramer et al. (2018) in the Web of Science, PubMed, and Scopus databases in November 2020. The question that guided the search strategy was: What are the characteristics of bystander roles in cyberbullying situations in adolescent students? To carry out the review, a combination of search terms were applied, including Medical Subject Headings (MeSH), for example, *bystander, cyberbystanders, adolescent* (MeSH), *student* (MeSH), *cyberbullying* (MeSH), *participant role*, and *bystander intervention*. From several articles (*n* = 233), those with a title related to the topic of interest (*n* = 98) were first screened followed by those having a relevant abstract (*n* = 47). Subsequently, the full articles were reviewed following the standard quality assessment criteria used to evaluate primary research articles (Kmet et al., 2004), and the inclusion and exclusion criteria were applied, resulting in nine articles being selected (Figure 1).

### Inclusion Criteria

Articles that reported an identification and/or characterization of the types of bystanders in cyberbullying were included. Only scientific articles published in English or Spanish between the years 2015 and 2020 that included samples of adolescents were considered for the review.

### Exclusion Criteria

Articles that only addressed one type of bystander or that referred to this role in a general way were excluded. Articles that addressed traditional bullying, violence, or aggression between school children or cyberbullying in preschool children, elementary school children, or adults were excluded. Theses, conference proceedings, systematic reviews, or articles in a language other than English or Spanish or whose publication date preceded 2015 were not considered for the review.

## Results

Nine high-quality investigations were analyzed (Table 1), mostly representing cross-sectional studies. Due to their design, it was evident that the objectives, study design, sample selection method, measurement instruments, data analysis methods, and results were rigorously described and were appropriate in each of the selected studies.

**Table 1:** Types of cyberbystanders and related factors.

**References**	**Country(s), sample, age/grade**	**Results: Types of cyberbystander**	**Results: Related factors**
DeSmet et al. (2016)	*n* = 1,979 (47.3% men; 54.7% women). Age: 12–15 years. Belgium.	1) Group of adolescents who show positive behavior (defenders, those who provide support and who report what happened to others to adults and peers) (44.9%). 2) Group of adolescents who show negative behavior (passive behavior, enjoy watching cyberbullying, and those who reinforce the aggressor's behavior) (55.1%).	1) Group of adolescents with positive behavior - Intention to behave in a positive way (increased by friendship bond) - Greater self-efficacy - Negative attitude toward passivity - Positive attitude to comfort the victim - Previous victimization experience - Younger adolescents in the age range - Less moral disengagement - Mothers aware of the activities of their children on the internet - School organizes daily information for students 2) Group of adolescents with negative behavior - Intention to behave in a negative way - Positive attitude toward the passive observation - Greater moral disengagement - Greater in males - Older adolescents in the age range - Difficulties with social skills, empathy, and problem-solving - Decreases in parent-school communication
Erreygers et al. (2016)	*n* = 2,309 (50.3% men; 49.7% women). Age: 9–17 years. Belgium.	1) Joins the aggressor (4.6%). 2) Helps the victim (42.3%). 3) Not involved (53.6%).	1) Group joining the aggressor - Less empathy - Older - More impulsive 2) Group that helps the victim - Greater empathy - Younger - Less impulsive - Previous experience of victimization by bullying. - There were no statistically significant differences according to sex.
González-Cabrera et al. (2019)	*n* = 5.036 (49.3% men; 50.7% women). Age: Two age groups; 10–14 years and 15–23 years. Spain.	1) Defender of the victim (54.6%) 2) Assistant of the aggressor (2.2%) 3) Reinforcer of cyberbullying (1.6%) 4) Outsider (22.7%) 5) Supporter of the victim (18.3%)	- Significantly higher number of men in the assistant of the aggressor and outsider groups - Higher number of women in the defender of the victim group
Machackova and Pfetsch (2016)	*n* = 321 (66% men; 44% women). Age: 12–18 years. Germany.	1) Support the victims 2) Reinforce the aggressors (does not indicate percentages)	1) Group that supports victims - Affective empathy (cognitive empathy was not significant) 2) Group that reinforces the victims - Normative beliefs about cyberbullying The sex and age variables did not present statistically significant relationships with the groups of cyberbystanders.
Olenik-Shemesh et al. (2015)	*n* = 1,094 (51.6% men; 48.4% women). Age: 9–18 years. Israel.	1) Passive bystander (55.4%) 2) Active bystander (44.6%)	1) Passive bystander group - Men - Younger ages in the established range - Less perceived social support from significant others (greater emotional and social loneliness). 2) Active bystander group - Greater presence of women
			- Older than passive bystanders - Greater perceived social support from significant others (less emotional and social loneliness).
Panumaporn et al. (2020)	*n* = 578 (41.7% men; 58.3% women). Age: 11–19 years. Thailand.	1) Adolescents who are willing to intervene or help the victims (34.6%). 2) Adolescents who ignore the cyberbullying situation observed (28%). 3) Adolescents who partake in cyberbullying (26.3%).	1) Group willing to intervene - Previous bullying experiences (directly or through close friends /family) - High level of attachment to parents - Women - The behavior of providing support to the victim depends on whether the norm of the group is to intervene or ignore - Higher self-esteem 2) Group that ignores cyberbullying - There is no previous victimization, nor experience as aggressors. - They perceive that the norm of the group is to ignore, so they do not provide help 3) Group that joins in cyberbullying - Positive attitude toward bullying and participation - Older - Less attachment to parents - Previous experiences in the role of aggressor in traditional bullying
Quirk and Campbell (2015)	*n* = 716 (24.6% men; 75.4% women). Age 12–18 years. Australia.	1) Assistants (4.4%) 2) Reinforcers (7.4%) 3) Outsiders (63.2%) 4) Defenders (25%)	1) Assistants - Higher percentage of men 2) Outsiders - Low percentage of males - More older adolescents in the role of outsiders compared to defenders. No clarity regarding the distribution of women.
Schultze-Krumbholz et al. (2018)	*n* = 849 (45.6% men; 52.7% women). Age: 11–17 years. Germany.	1) Outsiders (28.4%) 2) Aggressive defenders (9.5%) 3) Prosocial defenders (52.2%) 4) Assistants (2.8%)	1) Outsiders - Low probability of participating as a bully, victim, defender, or assistant - Communicates the observed situations to parents or peers 2) Aggressive defenders - More likely to inform their peers than their parents - Related to reactive aggression - Engage as bullies and/or victims 3) Prosocial defenders - They provide support to the victim - They communicate the observed cyberbullying situations to their parents - Younger than all other groups - Low levels of proactive aggression - High levels of cognitive and affective empathy, compared to outsiders - Higher percentage of men 4) Assistants - Higher percentage of men - Reactive aggression. - Low cognitive empathy Self-esteem was not considered relevant in any group. Age and sex were not significant when other variables were included in the analysis.
Song and Oh (2018)	*n* = 1,058 (52.8% men; 47.2% women). Age: 14–19 years. South Korea.	1) Defenders (30.5%) 2) Outsiders (60.7%) 3) Reinforcers (5.4%) 4) Assistants (3.3%)	1) Defenders (evaluated defensive tendency in general) - Low moral disengagement - Low antisocial conformity - No experience in the role of aggressor - Greater empathy - Scarce/negative relation with the aggressor - Greater perceived control of the situation of aggression - Absence of other bystanders Age and sex not significant for any of the groups.

*n, Sample size*.

The studies came from different countries (Belgium, Spain, Czech Republic, Thailand, Germany, Israel, and Australia). The mean sample size of the nine studies was 1,572.7, but there was high variability (SD = 1,444.33). The sample sizes were between 321 and 5,036 adolescent students. Participant ages ranged from 9 to 23 years with most between 12 and 17 years, since the objective of the review was to address adolescent bystanders. Regarding the sex of the participants, the study by Quirk and Campbell (2015) stands out because it had a much higher percentage of women (75.4%), compared to the other investigations which had distributions close to 50% between the sexes.

### Bystander Roles in Cyberbullying: How Many and Who

#### Studies That Identified Two Bystander Groups

The study by DeSmet et al. (2016) referred to two groups of bystanders: those who show positive behavior (44.9%), that is, who defend or support the victim or report the incident to adults or peers, and those who show negative behavior (55.1%), which is related to passive behavior or enjoying and/or reinforcing the behavior of the aggressor. Olenik-Shemesh et al. (2015) reported a passive bystander role that included those who did not get involved in the cyberbullying situation (55.4%) and an active bystander role (44.6%) that incorporated adolescents who supported the victim. On the other hand, Machackova and Pfetsch (2016) made a distinction between adolescents who supported the victims and those who reinforced the actions of the aggressor, but the study did not report the percentage of each role.

#### Studies That Identified Three Bystander Groups

The studies by Panumaporn et al. (2020) and Erreygers et al. (2016) noted the following distinction: adolescents willing to intervene or help the victims (42.3–34.6%, respectively); adolescents who ignore the cyberbullying situation (53.6–28%); and finally, bystanders who join the cyberbully (4.6–26.3%) (Erreygers et al., 2016; Panumaporn et al., 2020).

#### Studies That Identified Four Bystander Groups

These studies showed a similar distribution and percentage of adolescents in each of the roles. Specifically, the following trends were observed: outsiders, 63.2–60.7%; defenders, 25–30.5%; reinforcers, 7.4–5.4%; and assistants 4.4–3.3% (Quirk and Campbell, 2015; Song and Oh, 2018). Also, Schultze-Krumbholz et al. (2018) distinguished four sub-roles but made a differentiation in terms of the types of the defender, with aggressive defenders characterized by behaviors such as confronting the aggressor, activating others, and comforting victims (9.5%) and prosocial (52.2%), who are more likely to support the victim and inform their parents. The other bystander subtypes indicated are outsiders (28.4%) and assistants (2.8%).

#### Studies That Identify Five Bystander Groups

The study by González-Cabrera et al. (2019) had the most extensive classification, grouping the participants into five sub-roles: defender of the victim (54.6%), supporter of the victim (18.3%), outsiders (22.7%), cyberbullying reinforcer (1.6%), and assistants of the aggressor (2.2%). There was a distinction made between positive behavior in the case of those who defended the victim, those who interrupted the situation, and those who provided help, in contrast with adolescents who only supported the victim but did not stop the aggressor.

### Bystander Roles in Cyberbullying: Who and Why

Regarding the different roles of cyberbystanders and gender, three investigations observed a higher percentage of men in the groups that presented negative behaviors (Olenik-Shemesh et al., 2015; DeSmet et al., 2016), specifically, in the roles of assistants of the aggressor (Quirk and Campbell, 2015; Schultze-Krumbholz et al., 2018) and outsiders (González-Cabrera et al., 2019). Meanwhile, women tended to show an active role, providing help to the victim of cyberbullying (Olenik-Shemesh et al., 2015; Machackova et al., 2016; González-Cabrera et al., 2019; Panumaporn et al., 2020). On the contrary, other studies analyzed did not find gender differences (Erreygers et al., 2016; Machackova and Pfetsch, 2016; Schultze-Krumbholz et al., 2018; Song and Oh, 2018).

With regard to age, four investigations indicated that younger adolescents were more likely to carry out positive interventions in cyberbullying situations than older adolescents, who tended to show negative or passive behavior (Olenik-Shemesh et al., 2015; Quirk and Campbell, 2015; DeSmet et al., 2016; Erreygers et al., 2016). Consequently, two studies showed a higher percentage of older adolescents in the group of bystanders who joined the aggressor (Erreygers et al., 2016; Panumaporn et al., 2020). On the other hand, three of the nine investigations did not find differences between ages (Machackova et al., 2016; Schultze-Krumbholz et al., 2018; Song and Oh, 2018).

In relation to personal variables, these were reported to have a significant association with the different bystander roles and were present with greater frequency in the results obtained. It was shown that experiences of previous victimization, those experienced directly or indirectly through close reports, increased the interventions to provide help to the victim in a cyberbullying situation (DeSmet et al., 2016; Erreygers et al., 2016; Panumaporn et al., 2020). However, previous experiences in the role of aggressor were linked to bystanders who were willing to join the cyberbully (Panumaporn et al., 2020). Therefore, Song and Oh (2018) reported that defenders do not share experiences of this type. Another relevant variable in the research is the self-efficacy perceived by adolescents regarding their intervention in the cyberbullying situation (Olenik-Shemesh et al., 2015; DeSmet et al., 2016).

With regard to social skills, the reviewed studies showed that empathy levels were low in adolescents who showed negative behaviors (DeSmet et al., 2016), such as those in the roles of assistants to the aggressor and outsiders (Erreygers et al., 2016; Schultze-Krumbholz et al., 2018), specifically, an association was shown with cognitive empathy (Barlińska et al., 2018; Schultze-Krumbholz et al., 2018). In contrast, adolescents who were willing to intervene had higher empathy levels (Erreygers et al., 2016; Schultze-Krumbholz et al., 2018; Song and Oh, 2018). Machackova et al. (2016) reported that affective empathy predicts support for the victim, while cognitive empathy did not show significant results. Along the same line, another variable associated with bystander roles was moral disengagement, which was related to interventions that supported the victim, while the greater the moral disengagement, the more the passive behavior increased (DeSmet et al., 2016; Song and Oh, 2018).

## Discussion

The objective of this research was to identify and characterize the roles of cyberbullying bystanders in adolescent students. The results allowed the identification of nine articles (Table 1), whose participants were adolescents from different countries (Belgium, Spain, Czech Republic, Thailand, Germany, Israel, and Australia), that met the standard quality assessment criteria (Kmet et al., 2004). The findings of this study showed that bystanders are not a homogeneous group in terms of characteristics and behavior. Specifically, the selected studies identified between two to five types of bystanders. Although the investigations that identified two groups of bystanders (Olenik-Shemesh et al., 2015; DeSmet et al., 2016; Machackova and Pfetsch, 2016), represent a progress in the studies that consider them a completely homogeneous group, they are still considered too general and could limit the understanding of the particularities of bystander roles in cyberbullying (Patterson et al., 2017; Knauf et al., 2018; Wright et al., 2018).

Differences were established with adolescents who show negative behavior, since their actions may be oriented toward either ignoring the situation or joining the aggressor (Erreygers et al., 2016; Panumaporn et al., 2020). The classification of four types of cyberbystanders (assistants, reinforcers, outsiders, and defenders) stood out because the sizes of these groups were similar in the Republic of Korea (Song and Oh, 2018) and in Australia (Quirk and Campbell, 2015), and this classification of bystanders coincided with that of Salmivalli et al. (1996) in regard to traditional bullying. Another finding revealed that, in most of the studies reviewed, there were high percentages of adolescents who did not intervene in cyberbullying situations (Olenik-Shemesh et al., 2015; Quirk and Campbell, 2015; DeSmet et al., 2016; Erreygers et al., 2016; Song and Oh, 2018), contrary to research that shows that the highest percentage of adolescents assume the role of the spectator who intervenes, defends, and helps the victim (Schultze-Krumbholz et al., 2018; González-Cabrera et al., 2019). In this sense, the distinction between defenders who show prosocial behavior and defenders who manifest aggressive behavior stands out (Schultze-Krumbholz et al., 2018), showing that these students also assume other roles in cyberbullying such as those of victim or aggressor. This reflects that the internet favors less stable roles.

With regard to the different types of bystander roles and their characterization, it can be noted that several studies associated a higher percentage of males with roles that manifest negative or passive behavior, such as reinforcing or joining the aggressor (Olenik-Shemesh et al., 2015; Quirk and Campbell, 2015; DeSmet et al., 2016; Schultze-Krumbholz et al., 2018; González-Cabrera et al., 2019), while women tended to show behavior aimed at providing help to the cyberbullying victim (Olenik-Shemesh et al., 2015; Machackova et al., 2016; González-Cabrera et al., 2019; Panumaporn et al., 2020). This coincides with previous research on the role of bystanders, which associated women with greater prosociality (Allison and Bussey, 2017; Campbell et al., 2017; Patterson et al., 2017). Findings related to age were contradictory, with research showing that younger adolescents tended to carry out positive interventions in cyberbullying situations compared to older adolescents who showed negative or passive behavior (Olenik-Shemesh et al., 2015; Quirk and Campbell, 2015; DeSmet et al., 2016; Erreygers et al., 2016; Panumaporn et al., 2020), which could be explained as a desensitization effect (Pabian et al., 2016).

Along the same lines, the previous experiences that adolescents have had both in the role of victim and in the role of the aggressor are relevant (DeSmet et al., 2016; Erreygers et al., 2016; Song and Oh, 2018; Panumaporn et al., 2020), concurring with other investigations (Charaschanya and Blauw, 2017; Domínguez-Hernández et al., 2018; Kozubal et al., 2019; Huang et al., 2020). In the case of students in the spectator role with previous experiences as an aggressor, it is evident that they could continue to affect the lives of other students by supporting cyberbullying situations. This result shows the need to continue making efforts to curb the increase in cyberbullying in a highly digitized society. On the other hand, the self-efficacy perception of the adolescent and the belief that their actions can interrupt the cyberbullying situation arose as a factor addressed in the literature (Olenik-Shemesh et al., 2015; DeSmet et al., 2016), since adolescents who did not intervene had lower levels of social and emotional self-efficacy and therefore reported a higher perception of fear (Olenik-Shemesh et al., 2015).

Regarding social skills, many studies addressed empathy, noting that it was related to adolescents who intervened positively and with those who showed negative behaviors (DeSmet et al., 2016; Erreygers et al., 2016; Schultze-Krumbholz et al., 2018; Song and Oh, 2018). It is relevant to note that greater empathy is shown when the positive intervention involves prosocial and non-aggressive behaviors. It should also be taken into account that different investigations show a significant association specifically with cognitive empathy: in contrast, this relationship is not observed for affective empathy (Barlińska et al., 2018; Schultze-Krumbholz et al., 2018), although there is no agreement on this (Kozubal et al., 2019). It is also linked to sex, since women view cyberbullying situations as more serious and intervene more frequently, which could be explained by greater empathy (Huang et al., 2019; 2020). However, according to DeSmet et al. (2016), empathy is a less significant predictor in cyberbullying than in traditional bullying due to the lower emotional participation of students linked to the characteristics of digital platforms. In turn, greater moral disengagement was associated with the different bystander roles that exhibit passive behavior (DeSmet et al., 2016; Song and Oh, 2018). Coincidentally, studies have linked difficulties in the development of social skills with cyberbullying, observing that moral disengagement is related to problems in evaluating the situation they are witnessing (Domínguez-Hernández et al., 2018; Knauf et al., 2018; Antoniadou et al., 2019).

It should be noted that the efforts to identify and characterize the types of bystanders were aimed at generating a greater understanding of who, how many, and why adolescents belong in each of the bystander groups. Achieving greater knowledge in the area will allow better planning and development of interventions, considering that bystanders do not represent a group with homogeneous characteristics. Thus, it is relevant to include distinctions between spectators related to personal and social variables that transcend the binary categorization of active/passive behavior or positive/negative behavior. It is also necessary to integrate contextual variables, transitioning from individual to social (Gálvez-Nieto et al., 2020), since cyberbullying is a phenomenon that requires a multidisciplinary approach.

## Author Contributions

All authors listed have made a substantial, direct and intellectual contribution to the work, and approved it for publication.

## Conflict of Interest

The authors declare that the research was conducted in the absence of any commercial or financial relationships that could be construed as a potential conflict of interest.
